# Psychological Dimensions of AI Implementation in Taekwondo: A Qualitative Case Study of Coach and Athlete Perceptions

**DOI:** 10.3390/bs16081377

**Published:** 2026-08-11

**Authors:** Erol Baykan, Rouba Jamal Eddine, Ergun Gide

**Affiliations:** 1 Department of Sports Management, Faculty of Sport Sciences, Yozgat Bozok University, 66100 Yozgat, Turkey; erol.baykan@yobu.edu.tr; 2 School of Engineering and Technology, Central Queensland University, Sydney, NSW 2000, Australia; e.gide1@cqu.edu.au

**Keywords:** self-efficacy, self-determination theory, AI acceptance, human oversight, qualitative content analysis, sport technology, responsible AI, athlete monitoring, referee decision support, coach–athlete relationship, UN-SDG 9

## Abstract

This qualitative case study examined how university taekwondo coaches and athletes perceive artificial intelligence (AI) in sport, with emphasis on the psychological dimensions of implementation readiness. Using convenience sampling, semi-structured interviews were conducted with 23 coaches and 30 athletes competing in inter-university taekwondo championships in Turkey. Data were analysed through qualitative content analysis, and inter-coder agreement was quantified using Cohen’s Kappa. Competition analysis and training support were identified as the most useful applications. Reported benefits included faster feedback, improved athlete monitoring, and more systematic performance analysis, while concerns centred on incorrect guidance, loss of human elements in coaching, and insufficient accountability in AI-supported decisions. Athletes were generally positive about AI-assisted refereeing when it was framed around fairness and visibility, whereas coaches were more cautious when AI was presented as a substitute for core coaching functions. Interpreted through self-determination theory and self-efficacy as sensitising lenses, the findings indicate that AI adoption is most feasible when systems support rather than displace human roles, operate under clear governance and human oversight, and are accompanied by targeted training. The findings are context-specific and provide a basis for further empirical work. The findings of this study contribute to UN-SDG 9 by supporting the responsible adoption of artificial intelligence in sport management.

## 1. Introduction

Artificial intelligence (AI) is commonly framed as a family of computational methods that enable systems to perceive, learn from data, and support decision-making under uncertainty (Garg, 2021; Russell & Norvig, 2010). As the sports industry undergoes rapid technological change, AI has increasingly been positioned as an enabling capability across training, competition analysis, and practitioner decision support (Hammes et al., 2022; Sampedro, 2023). In contemporary sport settings, data obtained during competitions and training sessions can be analysed to inform athlete development and team strategies, contributing to more systematic and data-driven decision processes. Within this context, AI-based tools have been proposed to support coaches operating in time-constrained, high-stakes environments, where decision quality is shaped by context-specific constraints (Aarons et al., 2024).

AI methods have also been applied to core sports-analytics tasks. Performance prediction has been explored using machine learning models trained on team and match features (McCabe & Trevathan, 2008). In parallel, image and signal processing approaches have been widely reported across elite sports for extracting tactical and performance-relevant indicators from video, sensor, and wearable data, while modelling and planning approaches have gained increasing attention for decision support (Hammes et al., 2022). More broadly, advances in machine learning and deep learning have strengthened capability in perception and pattern extraction tasks that underpin many applied sports-analytics pipelines (Goodfellow et al., 2016).

Taekwondo is a martial art that requires fundamental motor skills and diverse physical attributes, and effective training approaches are required to optimise performance (Karademir et al., 2022). AI techniques can support the analysis of taekwondo techniques through the observation of match videos and motion patterns, supporting the identification of strengths, weaknesses, and training targets (Shin et al., 2024). Similarly, AI-enabled analysis of athlete- and equipment-derived data has been discussed across the broader sports domain, including applications related to efficiency improvement, injury risk estimation, and outcome-related insights (Li, 2021). Work synthesising AI use in martial arts highlights applications such as movement recognition, posture prediction, movement evaluation, and athlete support, suggesting a contribution to more data-driven training methodologies (Pang et al., 2024). However, taekwondo competition analysis has been reported as labour-intensive in practice because key events may be manually recorded by researchers, extending analysis time and contributing to variability in scale and accuracy (Shin et al., 2024).

Technical feasibility alone does not determine adoption. Implementation in applied sport settings depends on whether practitioners perceive AI as useful, trustworthy, and consistent with their professional identity and relational expertise (Sperlich et al., 2023; Wachholz et al., 2025). This issue is particularly important in taekwondo because much of the emerging AI literature in martial arts remains oriented towards technical capabilities such as movement recognition, pose estimation, motion capture, and performance modelling, whereas less attention has been given to how coaches and athletes interpret these tools in relation to training practice, competition analysis, coaching authority, fairness, and human judgement. A stakeholder-centred study is therefore necessary because AI systems that are technically promising may still be resisted, misused, or only partially adopted if intended users regard them as opaque, unreliable, threatening to role identity, or insufficiently accountable in competitive contexts.

These considerations align with the concept of psychological readiness for AI implementation, understood here as an interpretive, qualitative lens for examining whether stakeholders perceive AI applications as understandable, trustworthy, and supportive of, rather than threatening to, human-centred practice. Psychological readiness complements, but is distinct from, purely technical or organisational readiness and is shaped by trust, perceived risk, and the perceived legitimacy of AI-supported decisions (Kim et al., 2025). In this study, self-determination theory (Ryan & Deci, 2000) and self-efficacy (Bandura, 1977) are used only as sensitising theoretical lenses. They guided interpretation of readiness-related meanings in the interview data; they were not directly measured, operationalised as variables, or tested as explanatory models. Accordingly, autonomy, competence, relatedness, confidence, and perceived capability were interpreted abductively after data collection from participant accounts concerning control, professional capability, coach–athlete relationships, AI literacy, trust, risk, and willingness to rely on AI under human oversight.

Recent empirical work has begun to move the AI-in-sport literature beyond general technological promise towards more specific questions of quality, trust, perceived suitability, and human oversight. A recent narrative review of AI in sport identifies expanding applications across biomechanics, performance enhancement, sports medicine, health monitoring, coaching, and talent identification, while also emphasising the continuing need to address implementation challenges and responsible use (Zhou et al., 2026). Evidence from exercise and training contexts further indicates that AI-generated training outputs should not be treated as automatically reliable. For example, expert evaluations of ChatGPT-generated running plans found that output quality improved when more detailed input information was provided, but the plans were not rated as optimal and were not recommended without expert coach feedback (Düking et al., 2024). Similarly, a pilot study of recreational athletes found that users of AI-generated training plans reported higher trust than non-users, while experienced coaches were not always able to distinguish AI-generated plans from human-developed plans (Wachholz et al., 2025). These findings suggest that AI capability, user trust, and professional judgement are increasingly interdependent in sport training contexts.

Recent research has also begun to examine the perceived suitability of AI coaches and GPT-based tools for coaching practice. Dindorf et al. investigated psychological and perceptual factors associated with AI-driven sports coaches and framed such systems as potentially augmenting, rather than simply replacing, human coaching in targeted training-support domains (Dindorf et al., 2025). O’Brien and Prentice further showed that freely available GPT tools could support coach learning and athlete-development analysis by rapidly extracting patterns from coach–athlete conversational data, while also identifying risks related to contextual misunderstanding, verification burden, and privacy (O’Brien & Prentice, 2025). In taekwondo specifically, Zhang et al. reported that an AI-supported video review approach using ChatGPT-4.5 and OpenPose showed strong agreement with international video review referees and substantially reduced review time in Paris Olympic taekwondo cases, while still concluding that human oversight remains necessary for ambiguous cases (Zhang et al., 2025). Collectively, this recent literature supports the need for stakeholder-centred studies that examine not only whether AI can technically support sport, but also how coaches and athletes perceive its benefits, risks, role boundaries, and conditions for responsible implementation.

However, the existing evidence remains uneven. Much of the literature evaluates AI systems, generated training plans, or broad sport-technology adoption, while fewer studies examine how martial-arts stakeholders themselves interpret AI in relation to coaching authority, athlete trust, competition analysis, and referee decision support. This gap is especially important in taekwondo, where human judgement, coach–athlete relationships, tactical interpretation, and perceptions of fairness are central to the competitive environment. The present study therefore addresses this gap by examining coach and athlete perceptions of AI within a bounded university taekwondo championship context and by interpreting these accounts through readiness-related psychological lenses rather than by claiming to measure psychological readiness as a validated construct.

Within this scope, the aim of this study is to examine how university taekwondo coaches and athletes perceive potential AI applications in training, competition analysis, coaching, and refereeing, and to interpret readiness-related perceptions abductively through these qualitative sensitising lenses. The study does not empirically test self-determination theory, self-efficacy, or formal hypotheses; instead, it is guided by the following qualitative research questions:

1. RQ1. How do university taekwondo coaches and athletes perceive the potential benefits and drawbacks of AI for training performance and athlete development?
2. RQ2. How do university taekwondo coaches and athletes perceive the potential benefits and drawbacks of AI for competition analysis?
3. RQ3. How do coaches perceive the implications of AI for the future coaching role, and how do athletes perceive AI-supported referee decision-making?
4. RQ4. What readiness-related perceptions, including perceived knowledge, trust, competence, autonomy, role security, fairness, accountability, and human oversight, shape acceptance of or resistance to AI in this taekwondo context?

This paper makes the following contributions. First, it provides empirical evidence on AI perception patterns among competitive taekwondo coaches and athletes across multiple application domains. Second, it develops a qualitative interpretation of readiness-related perceptions, using self-determination theory and self-efficacy as sensitising lenses rather than claiming to measure or definitively evaluate psychological readiness. Third, it proposes a conceptual synthesis map that organises AI application domains according to participant-perceived benefit, perceived risk, and readiness-related indicators, treated as interpretive patterns rather than measured constructs. Fourth, it offers a role-comparative description of perceptions while retaining cautious descriptive gender breakdowns where they inform AI-literacy considerations. Fifth, it offers a stakeholder-centred perspective on AI integration in martial arts contexts that complements the predominantly technical orientation of the existing taekwondo AI literature.

This paper is structured as follows. Section 2 reviews related work on AI in sports and technology acceptance. Section 3 describes the research methodology. Section 4 presents findings from the qualitative analysis. Section 5 discusses the results. Section 6 provides recommendations, including limitations (Section Limitations), and Section 7 concludes the study.

## 2. Related Work

### 2.1. AI Applications in Sports and Martial Arts: Technical Development and Stakeholder Acceptance

The integration of AI into sport has progressed from isolated experimental deployments to increasingly diverse application portfolios documented in narrative and scoping reviews (Hammes et al., 2022; Munoz-Macho et al., 2024). Within martial arts and taekwondo, recent work has described technical pathways that combine data capture and modelling with immersive training and analysis environments. For example, taekwondo-focused research has reported the use of three-dimensional reconstruction approaches, including 3D scanning and motion capture, to reconstruct athlete movements in 3D space and support training feedback and programme design, alongside metaverse (XR) environments intended to recreate competition-like settings (Shin et al., 2024). In parallel, surveys of AI in martial arts have synthesised applications such as action recognition, pose estimation, and action evaluation, positioning these capabilities as enabling technologies for performance analysis and athlete support (Pang et al., 2024). Collectively, this strand of literature evidences a broadening technical toolkit for sensing, modelling, and decision support in martial-arts contexts (Pang et al., 2024; Shin et al., 2024).

However, technical capability does not necessarily translate into adoption, and the literature repeatedly highlights the centrality of stakeholder trust, perceived usefulness, and perceived risk in shaping acceptance. SWOT-oriented analyses of AI in sport science emphasise that AI deployment may have deleterious social and psychological implications, including performance anxiety, pressure to conform to AI-generated recommendations, and reduced trust in coaching relationships, and further argue that resistance to AI adoption should be evaluated using technology-acceptance frameworks (Sperlich et al., 2023). Empirical evidence also indicates that acceptance and trust may differ substantially between users and non-users of AI-based tools. In a pilot study of recreational athletes, over half of participants reported using structured training plans, and a subset of these reported using AI-generated plans; users demonstrated significantly higher trust in these technologies than non-users, and expert coaches were often unable to reliably distinguish an AI-generated plan from a human-designed plan (Wachholz et al., 2025). These findings reinforce the position that implementation trajectories depend not only on model capability, but also on socio-technical factors that shape confidence, legitimacy, and willingness to rely on AI outputs (Sperlich et al., 2023; Wachholz et al., 2025).

In addition, domain-specific constraints can influence feasibility and uptake. For instance, taekwondo match analysis has been described as requiring manual event input in some settings, which increases analysis time and can introduce inconsistency across analysts and coding approaches (Shin et al., 2024). At the level of elite sport systems, scoping evidence characterises teams as complex, adaptive environments and highlights the practical need for well-structured datasets to support analysis workflows that inform performance and decision-making (Munoz-Macho et al., 2024). Taken together, the literature indicates that stakeholder-centred evaluation is essential to complement technical progress, particularly in contexts where data pipelines, operational constraints, and trust relationships interact (Munoz-Macho et al., 2024; Sperlich et al., 2023).

### 2.2. Responsible AI Adoption Considerations: Consolidating Evidence and Addressing Practical Gaps

Beyond demonstrating technical feasibility, the implementation of AI in sport requires governance and readiness considerations that address risk, accountability, and ethical constraints. Recent scoping work synthesises ethical issues in sport-facing AI applications, highlighting recurring concerns related to fairness and bias, transparency and explainability, athlete privacy and data ethics, and accountability for AI-supported decisions (Kim et al., 2025). Complementary SWOT-based perspectives emphasise that adoption pathways should anticipate potential harms and unintended consequences within athlete–coach ecosystems, and they foreground the role of structured acceptance assessment to manage resistance and sustain legitimate use (Sperlich et al., 2023). Narrative reviews further position AI as a set of enabling methods across perception, modelling, optimisation, and interaction, while simultaneously underscoring that real-world translation involves non-trivial operational and socio-technical challenges (Hammes et al., 2022).

Within martial arts, and taekwondo in particular, much of the published work remains oriented towards system and pipeline development, with comparatively less emphasis on empirically grounded implementation guidance that incorporates stakeholder perceptions across application domains (Pang et al., 2024; Shin et al., 2024). Moreover, evidence from adjacent sport contexts suggests that trust and intention to use can vary meaningfully between user groups, reinforcing the need to examine adoption considerations in a manner that is sensitive to stakeholder heterogeneity (Wachholz et al., 2025). Implementation-focused synthesis in elite sport also indicates that practical value depends on aligning analytic outputs with the dynamics of real operating environments and on ensuring that data organisation supports actionable interpretation (Munoz-Macho et al., 2024). Accordingly, the present study does not treat implementation readiness as a directly measured outcome; instead, it uses stakeholder perceptions to identify readiness-related considerations connected with legitimacy, usability, ethical acceptability, and psychological readiness (Kim et al., 2025; Sperlich et al., 2023).

This study responds to these needs by examining coach and athlete perceptions across multiple AI application domains in competitive taekwondo and by translating the resulting evidence into a conceptual synthesis that organises adoption considerations according to perceived value, perceived risk, and readiness-related concerns. In doing so, the study contributes stakeholder-centred evidence intended to complement the predominantly technical orientation of existing martial-arts AI literature and to support more responsible and context-sensitive adoption pathways (Pang et al., 2024; Shin et al., 2024).

## 3. Method

### 3.1. Research Ethics Approval

Ethics approval for the research was obtained from the Yozgat Bozok University Social and Human Sciences Ethics Committee on 16 April 2025 (decision number 24/19).

### 3.2. Research Design and Participants

Within a qualitative research framework, an embedded single-case study design was adopted to examine the views of university coaches and athletes regarding AI as an emerging digital capability in sport contexts. In this study, the bounded *case* is defined as the Turkish inter-university taekwondo championship competition system during the 2024–2025 competitive cycle. This system was treated as one case because the participants competed within the same regulated championship environment, with shared competition rules, performance demands, qualification conditions, and decision contexts. The individual universities were therefore not conceptualised as separate institutional cases, since the study did not compare university policies, team cultures, or institutional implementation strategies. Instead, universities functioned as recruitment sites within the same championship system, and coaches and athletes were treated as embedded units of analysis within that bounded case. This framing is consistent with multi-site embedded case study designs in which the case is the shared institutional or practice setting and participants from multiple constituent units contribute to the contextualised understanding of how that case operates (Chmiliar, 2010; Hancock & Algozzine, 2006). In contrast to experimental approaches that prioritise controlled manipulation and hypothesis testing, case studies are frequently used to generate detailed descriptions and interpretive insights grounded in real-world conditions (Hancock & Algozzine, 2006). In qualitative inquiry, case study designs support comprehensive examination of the phenomenon within its context, enabling the identification and interpretation of salient conditions, processes, and stakeholder perspectives (Yıldırım & Simsek, 1999). Recruiting participants from multiple universities was therefore consistent with the single bounded case: it broadened the range of coach and athlete perspectives while preserving the common championship context as the analytical boundary. The generalisability of findings beyond this bounded system is not claimed.

The research involved voluntary participation by 23 coaches (10 female and 13 male), all university taekwondo team coaches, and 30 athletes (14 female and 16 male), all university student taekwondokas. Eligible participants were coaches responsible for university taekwondo teams and student-athletes who were members of university taekwondo teams competing in the inter-university championship context. Recruitment took place among teams accessible to the researcher during the championship setting. Coaches and athletes from multiple universities were approached when they were available, informed about the purpose of the study, and invited to participate voluntarily. Convenience sampling was used because participants were selected from those who met the eligibility criteria, were present in the championship context, were accessible to the researcher, and agreed to be interviewed; the sampling was not random, stratified, or designed to represent universities proportionally. The number of individuals who were approached, who declined participation, or who were excluded after initial contact was not systematically recorded; therefore, a response rate cannot be calculated, and this is acknowledged as a sampling-process limitation. Participants were recruited from multiple universities competing within the same championship context, rather than from a single institution. Sample size was not determined by an a priori power calculation since the study is qualitative and interpretive in orientation. Given the focused aim, specificity of the bounded case, and depth of cross-domain responses, the achieved sample was judged to provide sufficient information power for an exploratory qualitative case study (Malterud et al., 2016). Demographic characteristics of coach participants are presented in Table 1, and demographic characteristics of athlete participants are presented in Table 2.

### 3.3. Data Collection and Analysis

Data were collected using the semi-structured interview technique, which combines a predefined set of guiding questions with flexibility to probe, clarify, and explore participant responses in depth (Karasar, 1995).

Of the eight questions prepared for coaches, four collected demographic information (age, gender, coaching duration, and university), and four were open-ended interview questions: (1) Do you have knowledge about AI? If so, at what level? (2) What types of benefits and drawbacks might AI have for training performance? (3) What types of benefits and drawbacks might AI have for competition analysis? (4) What types of benefits and drawbacks might AI have for the future of the coaching role?

Of the nine questions prepared for athletes, five collected demographic information (age, gender, years of athletic experience, class year, and university), and four were open-ended interview questions: (1) Do you have knowledge about AI? If so, at what level? (2) What types of benefits and drawbacks might AI have for training performance? (3) What types of benefits and drawbacks might AI have for competition analysis? (4) What types of benefits and drawbacks might AI have regarding referee decisions?

The interview protocols for coaches and athletes were intentionally asymmetric in their final question. Coaches were asked about the future of the coaching role because this domain pertains most directly to their professional identity and forward-looking practice. Athletes were asked about referee decisions because this domain bears most directly on the procedural fairness and visibility conditions they experience in competition. The asymmetry is therefore a deliberate stakeholder-aligned design choice rather than an oversight, although it does mean that cross-stakeholder comparisons on the coaching-role and referee domains are not available from this dataset; this constraint is reflected in the Limitations section.

While the semi-structured interviews were designed inductively to capture broad, lived experiences of AI, the subsequent thematic interpretation was informed by self-determination theory and self-efficacy as sensitising concepts. This abductive approach allowed the researchers to ground the findings in participants’ accounts while interpreting readiness-related meanings cautiously. Consequently, the interview guide was not designed to measure psychological readiness as a scaled metric. Instead, readiness-related perceptions were treated analytically as a cross-cutting interpretive category inferred from responses concerning AI knowledge, perceived benefits, perceived drawbacks, trust, role implications, fairness, accountability, and human oversight.

The interview forms were evaluated by expert academicians and taekwondo coaches and were revised based on feedback. Data collection took place primarily at the inter-university championship venues in quiet areas where participants could speak privately, and, where necessary, through online video calls for participants unable to meet in person. Prior to the interviews, participants were provided with a written information sheet describing the study aim, the voluntary nature of participation, the right to withdraw, and the management of anonymity, and written informed consent was obtained. Interviews were conducted in Turkish and lasted an average of 25–30 min. With participant permission, interviews were audio-recorded for verbatim transcription; where recording was not possible or was not preferred by the participant, detailed contemporaneous notes were taken and expanded immediately after the interview. Although the interview guide was short, sufficient depth was sought through flexible follow-up probes attached to each open-ended question, including “Can you give an example?”, “Why would this be useful or risky?”, “How might this affect training or competition practice?”, and “Would AI support or replace human judgement?”. The interviewer continued probing until the participant’s meaning, examples, perceived benefits and risks, and role-related implications were sufficiently clear for coding. Responses were transcribed by the first author and subsequently organised by question for content analysis. Coding, theme development, and reliability checking were conducted on the original Turkish-language transcripts before the English reporting text was prepared. Reporting drew on coded sample statements presented in Table 3, Table 4, Table 5, Table 6, Table 7, Table 8, Table 9 and Table 10 and illustrative paraphrased statements presented in Section 4. These statements were prepared from Turkish-language transcripts and coded response summaries by the first author, reviewed by a bilingual co-author, and cross-checked using machine translation as an auxiliary verification step; they are used to convey coded meanings rather than to reproduce verbatim participant wording. The use of Claude (Anthropic) for translation assistance with the English manuscript draft is disclosed in the acknowledgments; coding and analysis were not delegated to any large language model. As a limitation, an independent back-translation procedure was not undertaken. Decisions on idiomatic or culturally specific expressions were discussed between authors and resolved in favour of preserving the participant’s intended meaning, with caution against the smoothing of emotionally salient phrasing.

Because the tables report coded sample statements rather than extended interview extracts, the Findings section also includes anonymised illustrative paraphrased statements to show how the major coded meanings appeared in participant accounts. These statements synthesise the meaning of Turkish transcript segments and coded responses, are identified by anonymised participant labels for transparency, and should not be read as verbatim extracts or close word-for-word translations.

The interviewer was the first author, an academic working in sports management who has prior research experience in taekwondo. The interviewer was not in a supervisory or evaluative relationship with any participant. A small number of participants were known to the interviewer through prior professional contact at championship events; in these cases, the interviewer made the conditions of voluntary participation, anonymity, and the absence of any consequence for non-participation explicit at the outset of the interview, and the same interview protocol was used as for participants not previously known. Reflexivity discussions were held between authors during analysis to consider how prior familiarity with the championship setting and with taekwondo coaching practice may have shaped probing decisions and interpretation; these discussions are reflected in the framing of findings as context-specific and in the conservative interpretation of within-sample gender patterns.

After the interviews, transcripts were transferred to a computer environment, organised by question, and analysed through a staged qualitative content-analysis process. The stages were: familiarisation through repeated reading; initial manual coding using a shared hybrid deductive–inductive codebook; inductive refinement of codes generated from approximately one-third of the transcripts; theme condensation by grouping semantically related codes into sub-themes and higher-order themes; theoretical cross-reading through the sensitising lenses in Table 11; a limited participant credibility check of the thematic interpretation; and final synthesis across coded patterns, illustrative paraphrased statements, and the conceptual map. Two coders, the first author and an associate professor in training science, independently coded the full set of Turkish-language transcripts manually (without qualitative analysis software), and each response could receive multiple non-mutually exclusive codes. Disagreements were tabulated, discussed in a structured adjudication meeting, and, where needed, referred to the third author; Cohen’s Kappa was computed on the independently coded dataset using the procedure described in Section 3.4.

The analysis moved from discrete responses to broader interpretive themes by comparing the shared meanings, assumptions, and application domains linking individual codes, rather than simply listing fragmented categories. Thus, lower-level codes were treated as evidence for higher-order perceptions concerning benefit, risk, role change, fairness, accountability, and readiness. Codes were organised in Microsoft Excel, and frequency (*f*) and percentage (%) values were calculated only as descriptive indicators of within-sample thematic salience; they are not inferential statistics, prevalence estimates, or generalisable population-level measures. Missing or unclassifiable data were minimal and are reported transparently: where a participant did not provide a codable response to an open-ended question, this is shown in the relevant table as a “No response/Unclassifiable” category rather than being excluded, so that all 23 coaches and 30 athletes are accounted for within each domain. Repetitive statements were grouped without disrupting semantic integrity. The illustrative paraphrased statements presented in Section 4 use pseudonymous identifiers such as Coach-F*n*, Coach-M*n*, Athlete-F*n*, and Athlete-M*n* to preserve anonymity while indicating participant group and gender category.

**Figure 1:**
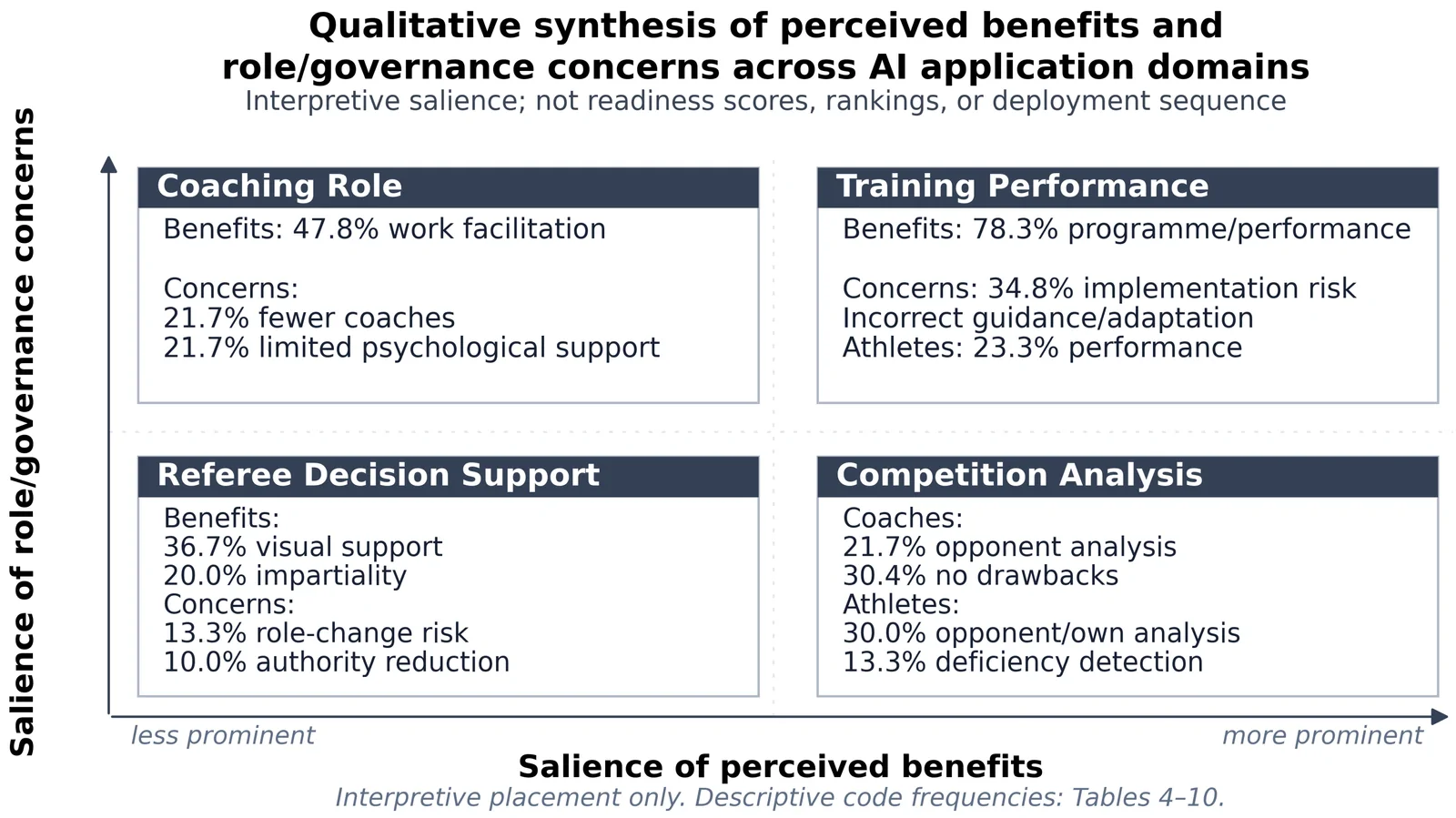
Thematic map of participant-perceived benefits and role/governance concerns across AI application domains in taekwondo. The horizontal axis summarises the salience of benefit-related themes in participant accounts, and the vertical axis summarises the salience of role-related and governance-related concerns. Both dimensions are interpretive and qualitative. The placement of each adoption domain reflects the relative prominence of these themes in participant accounts and should not be read as a measured score, a validated readiness rating, an implementation phase, or a deployment recommendation. Percentages, where shown, refer to descriptive code-frequency indicators reported in Table 4, Table 5, Table 6, Table 7, Table 8, Table 9 and Table 10; grouped-row percentages are component-code mention indicators rather than unique-participant proportions.

To support integrative interpretation, code frequencies were aggregated by application domain and used to inform a conceptual synthesis map (Figure 1). The map uses qualitative placement to summarise the relative salience of benefit-oriented, risk-oriented, and readiness-related themes. Distances and locations in the map should not be read as computed scores, validated readiness zones, or inferential comparisons.

### 3.4. Validity and Reliability

Various strategies were applied during data analysis to support validity, reliability, and reflexive transparency. Content validity of the semi-structured interview form was supported by consulting expert coaches and academicians prior to content analysis. During coding and theme and sub-theme generation, inter-coder consistency was assessed by comparing the independently produced coding outputs. As a limited credibility check, a summary of the main themes was discussed informally with a small subset of participants to assess whether the interpretations broadly reflected their intended meanings. This process was used as a plausibility check on the thematic interpretation rather than as a formal member-checking or participant-validation procedure. The analysis process was conducted openly and systematically, and themes, sub-themes, and sample statements were supported with frequency and percentage tables.

Researcher positionality was also considered as part of the validity strategy. The first author’s familiarity with taekwondo and inter-university championship settings provided contextual knowledge that helped the interviewer understand technical expressions, identify when follow-up probes were needed, and situate participant accounts within realistic training and competition practices. At the same time, this familiarity may have shaped the interview interaction and interpretation by making some taekwondo-specific assumptions appear self-evident or by encouraging more detailed probing in areas already familiar to the researcher. To manage this risk, the same interview protocol and probing prompts were used across participants, analytic decisions were discussed among authors, coding was checked by a second coder who was not positioned identically to the interviewer, and findings were framed conservatively as context-specific interpretations rather than as general claims about all taekwondo coaches and athletes.

Both coders used a shared coding framework (codebook) with clearly defined categories, decision rules, and exemplar statements. The codebook included AI-related categories aligned with the interview prompts, such as AI knowledge levels and perceived AI benefits and risks across training, competition analysis, coaching roles, and referee decisions. For the psychological readiness interpretation, these coded categories were then cross-read using the mapping in Table 11. Readiness was not coded as a standalone scale; rather, existing codes were interpreted as readiness-related evidence when they reflected perceived capability, trust, risk, autonomy, role security, relational support, fairness, accountability, or human oversight. Prior to full coding, the two coders conducted a brief pilot coding step on approximately one-fifth of the transcripts to align interpretation of the code definitions, discussed discrepancies, and refined category descriptions where needed.

Inter-coder consistency was determined using Cohen’s Kappa coefficient, computed as $\kappa =\left(P_{o}-P_{e}\right)/\left(1-P_{e}\right)$, where $P_{o}$ is the observed agreement and $P_{e}$ is the expected agreement. Cohen’s Kappa was considered appropriate for the present design because two coders independently judged the same set of transcript units and because each non-mutually exclusive code could be represented as a binary present/absent decision. This approach avoided forcing participant responses into single mutually exclusive categories, while still providing a chance-corrected index of agreement beyond simple percentage agreement. Because the coding scheme assigns multiple non-mutually exclusive codes per participant response within each coding domain, Kappa was computed at the participant-by-code binary judgement level rather than at the response level: for each code within a coding domain, each participant was assigned a 1 if the code was present in their response and 0 otherwise, and Kappa was calculated over the binary present/absent matrix produced by the two coders. The aggregate Kappa reported for each coding domain in Table 12 is the average of per-code Kappa values within that domain, weighted by the number of participant judgements contributing to each code. This procedure retains the multi-code structure of the qualitative analysis in the coding matrix, while acknowledging that Kappa itself evaluates agreement on simplified binary judgements rather than the full interpretive complexity of each response.

The Kappa results should therefore be read cautiously. First, for codes mentioned by only one or two participants, Kappa is unstable due to highly skewed marginals, and percent agreement is the more interpretable indicator at the individual-code level; the values reported in Table 12 should therefore be understood as domain-level summaries rather than as evidence of equivalent agreement across all codes within a domain. Second, averaging per-code Kappa values can mask variability between high-prevalence and low-prevalence codes, and the statistic does not resolve the interpretive judgement involved in deciding whether a nuanced statement expresses a given theme. Third, Kappa is sensitive to prevalence and marginal imbalance, which is especially relevant in multi-code qualitative datasets where some concerns or benefits are naturally rare. In future studies, Krippendorff’s alpha, prevalence-adjusted agreement indices, or the reporting of Kappa alongside percent agreement statistics computed from the full two-coder contingency matrix could provide a more comprehensive treatment of multi-code qualitative reliability. In the present study, Cohen’s Kappa was retained because the reliability assessment involved exactly two coders making binary code-presence judgements, and because it remains a transparent and commonly understood agreement statistic for two-coder content analysis. Kappa values are reported in Table 12 with interpretation bands following Landis and Koch (1977).

## 4. Findings

This section reports the findings through a thematic synthesis followed by detailed descriptive tables and illustrative paraphrased statements. Figure 1 provides an overview of the main qualitative patterns across AI application domains. The figure is heuristic and interpretive; it does not present measured readiness scores, implementation rankings, or inferential comparisons.

The thematic map shown in Figure 1 synthesises findings from Table 4, Table 5, Table 6, Table 7, Table 8, Table 9 and Table 10. The horizontal dimension summarises whether participant accounts emphasised concrete benefits more strongly, while the vertical dimension summarises the salience of role-related and governance-related concerns. To make the placement logic explicit, each domain was positioned by comparing, within that domain, the relative salience of benefit-oriented codes against role-related and governance-related concern codes, as reported in Table 4, Table 5, Table 6, Table 7, Table 8, Table 9 and Table 10. Domains in which benefit codes were raised more frequently and consistently than concern codes were placed further along the benefit dimension, and domains in which role or governance concerns were more prominent were placed higher on the concern dimension. No numerical scoring, weighting, or thresholding was applied; the positions express ordinal within-sample salience only and are not coordinates on a measured scale. These dimensions are interpretive and qualitative; they do not represent measured readiness scores, ranked priorities, or implementation phases. The map therefore highlights four broad patterns: competition analysis showed relatively higher benefit salience because participant accounts emphasised concrete benefits and role-displacement concerns were less prominent; training support showed a benefit-and-concern profile because programme-support benefits coexisted with guidance and adaptation concerns; referee decision support showed a benefit-and-concern profile because fairness and visibility benefits coexisted with authority and role concerns; and coaching-role AI showed greater concern salience due to identity, authority, psychological-support, and relationship themes. The overall pattern of readiness-related perceptions was therefore conditional: AI was regarded as acceptable when it augmented human roles and as less acceptable when it appeared to replace coach judgement, athlete agency, or referee authority.

The following demographic, knowledge-level, and domain-specific tables provide detailed descriptive evidence supporting this synthesis. Frequency tables are complemented by longer anonymised illustrative paraphrased statements so that the coded distributions are supported by richer qualitative evidence. Participant identifiers indicate role and gender category only; they do not correspond to names, institutions, or interview order. All gender-disaggregated values in this section are exploratory within-sample descriptors; the sample and subgroup cell sizes are too small to support inferential, psychological, or population-level claims about gender differences.

Coach participants comprised 43.5% female (n=10) and 56.5% male (n=13). Regarding coaching experience, 30.4% of participants had 0–5 years, 21.7% had 6–10 years, 26.1% had 11–20 years, and 21.7% had 21 years and above of coaching experience. In addition, 82.6% of participants were from different universities, while 17.4% were from the same university.

Athlete student participants comprised 46.7% female (n=14) and 53.3% male (n=16). Regarding athletic experience, 16.7% of participants had 0–5 years, 56.7% had 6–10 years, and 26.7% had 11 years and above of experience. In the class distribution, the highest proportion belonged to 1st year students at 56.7%. It was observed that 73.3% of participants were from different universities while 26.7% were from the same university.

Although age was collected during interviews, age values were not available in the anonymised dataset used for final analysis and reporting; therefore, the age mean and standard deviation cannot be reported. This is acknowledged as a demographic-reporting limitation.

**Figure 2:**
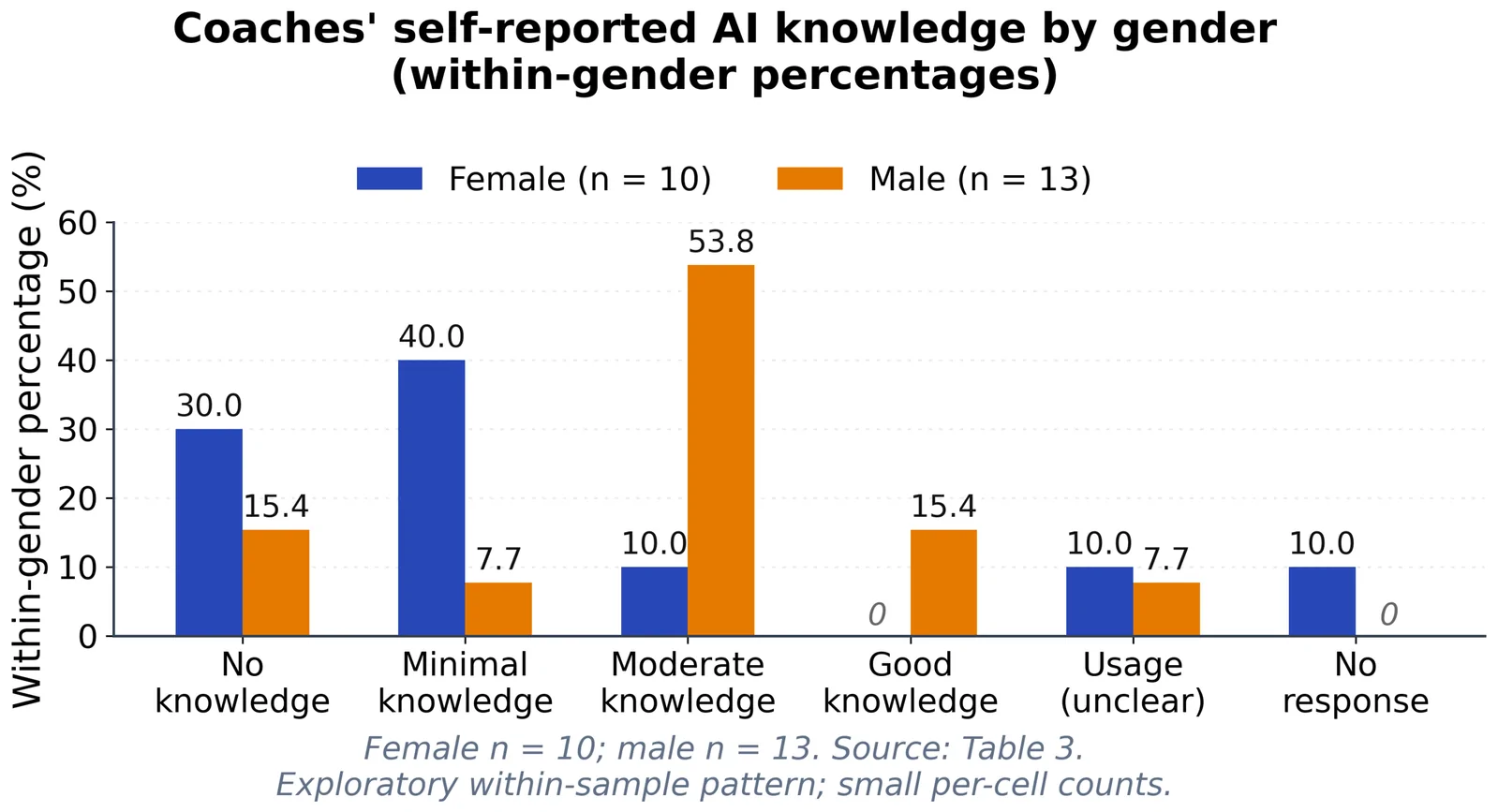
Exploratory within-sample gender distribution of coaches’ self-reported AI knowledge categories.

As shown in Figure 2 and Table 3, coaches’ self-reported AI knowledge varied across the sample. In this bounded sample, female coaches more often described no or minimal knowledge, whereas male coaches more often described moderate-to-good knowledge. These values are exploratory within-sample observations only; the small subgroup sizes do not support inferential, psychological, or population-level claims about gender differences. The illustrative paraphrased statements below show how perceived competence varied from basic awareness to uncertainty about practical application.

Illustrative paraphrased statement (Coach-F1): I have only minimal knowledge of AI. I have heard about it, but I have not yet explored how it works or how it could be used in taekwondo.

Illustrative paraphrased statement (Coach-M1): I have only learned about AI recently. I am still unsure how these tools would be applied practically during taekwondo training or competition preparation.

In Table 4, coaches primarily framed AI as a support for training programming, performance development, and data-informed planning. The main drawback pattern concerned implementation risks, especially incorrect programmes and reduced training discipline. Read alongside athletes’ training responses (Table 8), the table suggests a shared interest in AI as an augmentative planning aid, provided that coach judgement and contextual adaptation remain central. The following illustrative paraphrased statements show that coaches imagined AI as a data-supported planning aid, but not as a tool that should be followed uncritically.

Illustrative paraphrased statement (Coach-M2): One of the main benefits is that AI can help prepare training programmes using data. This could make the coach’s work easier and support improvements in athlete performance.

Illustrative paraphrased statement (Coach-F2): AI could support data-based training by helping us track athlete information, monitor health indicators, and follow motor development more systematically.

Illustrative paraphrased statement (Coach-F3): My main concern is the possibility of an incorrect training programme. If athletes simply follow a machine, there may be a loss of discipline and a risk of laziness in training.

Illustrative paraphrased statement (Coach-M3): AI can process numbers, but it does not have the experience or emotional understanding of a coach. It may not recognise injury risks or fatigue in the same way an experienced coach can.

According to Table 5, coaches interpreted AI-supported competition analysis as useful when it offered technical and operational support, opponent pattern recognition, error detection, and performance data. The grouped pattern suggests a broader expectation that AI could make analysis more systematic rather than simply adding isolated technical functions. Drawbacks centred on implementation and competitive risks, particularly incorrect information, time required for use, and the possibility that opponents could use similar systems. The no-drawback or no-opinion responses indicate an acceptance-related pattern, but this should be interpreted cautiously because limited exposure to domain-specific failure modes may also have shaped these responses.

Illustrative paraphrased statement (Coach-M4): AI could be very useful for opponent analysis. It may help us identify patterns in an opponent’s fighting style that are difficult to see during the match.

Illustrative paraphrased statement (Coach-F4): AI could provide a more systematic competition analysis by detecting incorrect techniques and identifying errors that may happen too quickly for the human eye to notice.

Illustrative paraphrased statement (Coach-M5): There is a real risk if the system provides incorrect information. If the analysis is wrong and we build the match strategy around it, the athlete could be disadvantaged.

Illustrative paraphrased statement (Coach-F5): Even if AI provides data about speed, strength or performance, it may not be useful unless the coach can interpret that data correctly and apply it to training.

According to Table 6, coaches most clearly positioned AI as a work-facilitation tool that could support programme preparation, analysis, information provision, and professional development. Concerns were organised around relational and psychological limits, role security, and implementation risks. The illustrative paraphrased statements below show the central tension between AI as work facilitation and AI as a possible threat to coaching authority, employment, and relational practice.

Illustrative paraphrased statement (Coach-M6): At its best, AI can facilitate the coach’s work. It can support analysis, reduce some of the workload, and help with programme preparation.

Illustrative paraphrased statement (Coach-F6): My concern is about the future of coaching. If these systems begin to do too much, the number of coaches may decrease and the profession may be weakened.

Illustrative paraphrased statement (Coach-M7): I worry that the coach could be pushed into the background. If athletes start relying more on the system than on the coach, coaching authority may be reduced.

Illustrative paraphrased statement (Coach-F7): AI may provide a diet or training programme, but it cannot provide psychological support. It cannot motivate an athlete who is anxious or struggling before a match.

Illustrative paraphrased statement (Coach-M8): The athlete–coach relationship is central in taekwondo. If we rely only on algorithms, that relationship may weaken and the human connection may be lost.

Illustrative paraphrased statement (Coach-F8): AI does not have the emotional intelligence needed in this sport. A coach knows when to push an athlete and when the athlete needs rest or support.

According to Table 7, athletes’ self-reported AI knowledge was distributed across no, minimal, moderate, and good knowledge or regular-use categories. Within this sample, athlete responses were broadly similar across female and male participants. These values are exploratory within-sample observations only; the sample size is too small to support inferential, psychological, or population-level claims about gender differences. One illustrative paraphrased statement shows how practical exposure to generative AI could coexist with limited domain-specific expertise.

Illustrative paraphrased statement (Athlete-M1): I would describe my knowledge as moderate. I use tools such as ChatGPT for general purposes, so I understand the basic idea, but I am not an expert.

According to Table 8, athletes framed AI training support around performance enhancement, planning, personalisation, nutrition, and technical or professional support. Their concerns focused less on opposition to technology itself and more on whether AI guidance would fit individual needs, avoid unsafe or unproductive prescriptions, and remain trustworthy. Athletes’ accounts similarly framed AI training support as useful only when it remained individualised and safe.

Illustrative paraphrased statement (Athlete-F1): If AI can analyse my physical data and provide a programme that is appropriate for my training needs, I think it could help improve my performance.

Illustrative paraphrased statement (Athlete-M2): If AI gives incorrect guidance or a programme that does not match my actual physical condition, I may struggle to adapt to it, and it could even increase the risk of injury.

According to Table 9, athletes valued AI-supported competition analysis when it helped them understand opponents, recognise their own deficiencies, develop strategy, and receive more systematic evaluation. The grouped drawbacks show that this positive orientation coexisted with concerns about analytic reliability, information vulnerability, and practical relevance. The following illustrative paraphrased statements show that athletes valued competition analysis when it supported self-evaluation, opponent preparation, and strategic feedback.

Illustrative paraphrased statement (Athlete-F2): The biggest advantage for me would be learning my opponent’s strengths and weaknesses, while also seeing my own deficiencies more clearly.

Illustrative paraphrased statement (Athlete-M3): I would trust AI more if it provided accurate analysis and instant feedback that could help with strategy development.

According to Table 10, athlete support for AI-assisted refereeing centred on visibility, fairness, decision support, and perceived error reduction. Concerns were not primarily about visual technology itself, but about the possibility that AI could weaken human authority, reshape the refereeing role, or make match management less relational and contestable. These illustrative paraphrased statements show that support for AI-assisted refereeing was strongest when AI was framed as improving fairness and visibility, but weaker when it appeared to displace human authority, contestability, and accountability.

Illustrative paraphrased statement (Athlete-M4): I support AI in refereeing because it can help see things that referees may miss. The actions are very fast, and AI could identify points that the human eye does not catch.

Illustrative paraphrased statement (Athlete-F3): It could make the competition fairer by reducing bias. Athletes would feel more confident that one side is not being favoured.

Illustrative paraphrased statement (Athlete-M5): AI could make match management clearer and more transparent. It may also reduce referee errors.

Illustrative paraphrased statement (Athlete-F4): My concern is that AI could eliminate the refereeing role. We still need human referees to manage the flow and safety of the fight.

Illustrative paraphrased statement (Athlete-M6): If AI becomes too dominant, it could damage the spirit of the sport. Managing a match only through AI would be problematic.

Illustrative paraphrased statement (Athlete-F5): There still needs to be human authority and initiative. If a sensor fails or a situation is complicated, a human referee should be able to make the final decision.

Illustrative paraphrased statement (Coach-M9): I am open to AI if it is used as supportive analysis. It should contribute to coach development and make our work easier, not replace the coach.

Illustrative paraphrased statement (Athlete-F6): AI can provide scientific data and technical support, but it should not replace the experience, judgement and emotional guidance of my coach.

Illustrative paraphrased statement (Coach-F9): For this to work safely, we need secure analysis and clear rules. Otherwise, information could be misused, and coaches may not trust the system.

Inter-coder reliability is reported separately in Table 12 (interpretation bands following Landis & Koch, 1977).

## 5. Discussion

This study examined how university taekwondo coaches and athletes interpreted possible AI applications in training, competition analysis, coaching, and refereeing. Rather than restating each coded frequency, the discussion is organised around the four research questions and three higher-order themes: AI as an augmentative support for performance work, trust and accountability in AI-supported decisions, and preservation of the human relationships through which coaching and competition acquire legitimacy. Gender-disaggregated observations remain exploratory within-sample descriptors only and are not interpreted as inferential or population-level evidence.

The findings are consistent with recent AI-in-sport research showing that AI adoption is shaped less by technical capability alone than by perceived trustworthiness, contextual adequacy, and the preservation of human expertise. In training contexts, participants in the present study viewed AI most positively when it was framed as support for programme design, feedback, monitoring, and performance analysis. This aligns with evidence that AI-generated training plans may become more useful when richer contextual input is provided, but still require expert interpretation and coach feedback (Düking et al., 2024). It also aligns with findings that trust differs between users and non-users of AI-generated training plans, indicating that acceptance is shaped by prior exposure and perceived usefulness rather than by system availability alone (Wachholz et al., 2025). The present findings therefore support an augmentation-based interpretation: AI may strengthen coaching decisions when it helps organise information, identify patterns, and accelerate feedback, but readiness is weaker when AI is perceived as replacing professional judgement or reducing athlete discipline.

### 5.1. RQ1: Training Performance, Athlete Development, and AI Capacity Building

For RQ1, coaches and athletes converged around a broadly positive but conditional view of AI in training. Coaches tended to frame AI as useful for programme preparation, monitoring, performance improvement, and data-supported decision-making; athletes emphasised personalised training, performance enhancement, and feedback. This pattern is consistent with AI-in-sport reviews that position AI as a tool for perception, modelling, optimisation, and decision support across training environments (Hammes et al., 2022; Munoz-Macho et al., 2024). It also aligns with athlete-focused evidence showing that AI coaching tools may be valued for personalisation and efficiency while still raising concerns about privacy, technical limitations, and reduced human contact (Ghezelseflou & Choori, 2023). In the present study, therefore, perceived usefulness was strongest when AI was imagined as a support for coach and athlete judgement rather than as a replacement for situated training expertise.

This distinction between augmentation and substitution is important for interpreting technology acceptance in sport. The participants’ positive responses were not expressions of unqualified technological optimism; rather, they were tied to concrete use cases in which AI could organise information, accelerate feedback, or help tailor preparation. Such a pattern is compatible with sport-AI SWOT analyses that identify efficiency and optimisation as opportunities while warning that adoption may be undermined by over-dependence, privacy exposure, and pressure to conform to algorithmic recommendations (Sperlich et al., 2023). The present findings therefore add a taekwondo-specific example of a broader acceptance condition: AI appears more legitimate when it improves the information environment around practice while leaving interpretive responsibility with coaches and athletes.

The main reservations in the training domain concerned incorrect guidance, poor fit to the athlete’s condition, over-reliance, laziness, discipline loss, and uncertainty about whether AI outputs could be trusted. These concerns echo technology-acceptance and sport-AI literature in which adoption depends not only on technical capability but also on perceived risk, prior exposure, confidence, and willingness to rely on system outputs (Sperlich et al., 2023; Wachholz et al., 2025). The self-reported AI knowledge results reinforce this point. Coach knowledge showed an exploratory gender pattern, whereas athlete knowledge appeared more similar by gender; given the small cell sizes, the practical implication is not subgroup labelling but broad, accessible capacity building. Comparable work on digital literacy and AI awareness suggests that perceived competence can vary across educational and demographic contexts (Gültekin & Özel, 2024; İçöz & İçöz, 2024). In taekwondo, structured demonstrations, guided interpretation of AI outputs, and explicit discussion of limitations may therefore be as important as the tools themselves.

### 5.2. RQ2: Competition Analysis, Interpretability, and Trust

For RQ2, competition analysis emerged as the lowest-resistance application domain. Both coaches and athletes saw value in opponent analysis, personal performance review, error detection, strategy development, and more systematic use of match data. This finding extends the technical literature on martial-arts AI, which has emphasised action recognition, pose estimation, movement evaluation, and taekwondo-specific video or motion-analysis pipelines (Pang et al., 2024; Shin et al., 2024). It also corresponds with broader elite-sport evidence that AI can support analysis workflows when data are organised in ways that produce actionable interpretation for practitioners (Munoz-Macho et al., 2024). The contribution of the present study is to show that stakeholders may be receptive to these analytic functions because they fit existing preparation practices and do not immediately threaten the coach–athlete relationship.

The competition-analysis findings also clarify where stakeholder perceptions complement the predominantly technical martial-arts literature. Existing work demonstrates that AI can classify, reconstruct, or evaluate movement patterns, but it infrequently addresses how coaches and athletes would use those outputs in preparation and tactical reasoning (Pang et al., 2024; Shin et al., 2024). Participants in this study treated analysis as valuable only when it could be translated into meaningful action: identifying tendencies, checking errors, adjusting strategy, or supporting post-match learning. Thus, interpretability should be understood not merely as model explainability, but as practical intelligibility within the routines of taekwondo training and competition review.

However, acceptance of competition analysis was not unconditional. Coaches worried that incorrect information could lead to flawed strategy, while athletes raised concerns about misleading feedback, ineffective analysis, and information vulnerability when opponents might access similar tools. These concerns sharpen the trust question: the issue is not simply whether AI can detect patterns, but whether users can understand the basis of an analysis, judge its reliability, and retain responsibility for tactical decisions. Work on coach decision-making in competitive sport similarly suggests that AI support must be aligned with the constraints and pressures under which coaches interpret information during matches (Aarons et al., 2024). Ethics-oriented reviews further emphasise transparency, explainability, privacy, and accountability as conditions for trustworthy sport-facing AI (Kim et al., 2025). The finding that some coaches saw no drawbacks in competition analysis may therefore indicate either close alignment with existing analytic routines or limited exposure to failure modes; future studies should probe how practitioners define acceptable error, evidence quality, and responsibility in this domain.

### 5.3. RQ3: Coaching Roles, Refereeing, and Human Oversight

For RQ3, the coaching-role and refereeing findings point to a common higher-order issue: AI is acceptable when it increases visibility or efficiency, but contested when it appears to displace human authority. Coaches did not reject AI categorically; many viewed it as a way to facilitate work, reduce workload, support analysis, or assist programme preparation. Their strongest concerns, however, involved professional role security, loss of authority, weakened psychological support, and erosion of the athlete–coach bond. These concerns are compatible with sport-AI discussions that describe opportunities for automation while warning that coaching is also relational, motivational, and interpretive (Sperlich et al., 2023). They also support the view that AI implementation in coaching should be treated as a socio-technical change process, not a purely technical upgrade.

This role-related concern is also consistent with research on coach decision support, which suggests that technology must account for the real-time, contextual, and socially embedded nature of coaching judgements (Aarons et al., 2024). A taekwondo coach does not simply select an optimal drill from a data profile; the coach also manages discipline, confidence, motivation, emotional regulation, and the timing of feedback. For this reason, systems that automate repetitive analysis may be welcomed, whereas systems framed as replacing the coach’s interpretive or relational work are likely to meet resistance. The coach–athlete relationship is therefore not an implementation detail but a core adoption condition.

The coach-role findings also correspond with recent work on AI-driven sports coaches and GPT-supported coach learning. Dindorf et al. suggest that AI coaches may be perceived as suitable for targeted training-support tasks, but such suitability depends on psychological and perceptual factors rather than on functionality alone (Dindorf et al., 2025). O’Brien and Prentice similarly show that GPT tools can provide efficiency gains for analysing coach–athlete dialogue, yet their usefulness depends on careful prompting, contextual understanding, verification, and privacy safeguards (O’Brien & Prentice, 2025). These studies help explain why coaches in the present study accepted AI most readily as a work-facilitating tool but expressed concern when AI was imagined as displacing core coaching functions. From a self-determination and self-efficacy perspective, this pattern suggests that AI implementation is more psychologically acceptable when it supports coaches’ perceived competence and autonomy, while preserving the relational dimensions of coaching.

Athletes’ views on AI-assisted refereeing were more favourable when the technology was framed around fairness, visual support, impartiality, and reduction of human error. Because the athlete and coach protocols were intentionally asymmetric, these referee findings cannot be treated as a direct role comparison with coaching-role responses. Even so, they highlight a parallel governance condition: participants supported AI where it could assist humans in seeing, checking, or explaining decisions, but expressed concern about role elimination, reduced referee authority, AI dominance, and ambiguous match management. This pattern aligns with ethical AI-in-sport literature that links legitimacy to transparency, accountability, contestability, and protection of athlete rights and wellbeing (Kim et al., 2025; Sampedro, 2023). In practical terms, the findings favour human-in-the-loop refereeing and coaching systems in which AI outputs are reviewable, responsibility remains visible, and final authority is not obscured by automation.

The athlete findings on referee decision support should also be read alongside recent taekwondo-specific evidence. Zhang et al. found that AI-supported video review in Paris Olympic taekwondo cases showed strong agreement with international referees and reduced review time, but the authors still recommended a hybrid model in which AI-assisted pre-review is followed by referee confirmation (Zhang et al., 2025). This is consistent with the present participants’ preference for fairness, visibility, and reduced human error, while also retaining concern about AI dominance and the weakening of human authority. The convergence between technical feasibility evidence and stakeholder perception suggests that AI-supported refereeing may be more acceptable when it is presented as transparent decision support rather than as full replacement of human judgement.

### 5.4. RQ4: Psychological Readiness and Human-Centred Adoption

For RQ4, the overall pattern is best interpreted as conditional readiness rather than general acceptance or rejection. Participants saw clear benefits in domains that made performance information more visible, timely, and systematic, especially competition analysis and selected training-support functions. Resistance intensified when AI touched autonomy, competence, relatedness, role security, fairness, or accountability. This supports the psychological readiness lens introduced in Section 1 and mapped in Table 11: adoption depends on whether stakeholders perceive AI as understandable, trustworthy, competence-enhancing, and consistent with human-centred practice.

This conditional readiness also explains why the same technology category could generate both enthusiasm and caution. AI-supported training and analysis were attractive when they promised faster feedback and more systematic preparation, yet the same capabilities raised concern when participants imagined unverified recommendations, opaque decisions, or weakened interpersonal judgement. In this sense, trust should be viewed as situated and domain-specific rather than as a single attitude towards AI. This interpretation is consistent with ethical reviews arguing that sport-AI governance must address fairness, bias, privacy, explainability, and responsibility for AI-supported decisions together rather than treating trust as a purely individual preference (Kim et al., 2025; Sampedro, 2023).

Self-determination theory and self-efficacy help interpret these patterns as sensitising lenses, not as directly measured constructs. Coaches’ worries about being relegated to the background, losing discipline, or weakening psychological support can be read as perceived threats to autonomy, competence, and relatedness (Ryan & Deci, 2000). Knowledge gaps and uncertainty about AI outputs similarly point to perceived capability and confidence, which are central to self-efficacy theory (Bandura, 1977). This interpretation is consistent with technology-acceptance evidence that trust may be higher among users with exposure to AI-generated training plans than among non-users (Wachholz et al., 2025). Accordingly, readiness in this taekwondo context is likely to be strengthened through experiential AI literacy, scenario-based training, and opportunities for coaches and athletes to test system outputs against their own expertise.

These findings strengthen the rationale for the human-centred adoption-considerations framework proposed in Section 6. AI applications should be introduced as augmentative decision-support capabilities, with clear explanations of data sources, known limitations, privacy safeguards, and accountability routes. This is especially important in taekwondo because the most technically plausible applications identified in the literature—movement recognition, posture evaluation, competition analysis, and performance modelling—intersect with social practices built on trust, authority, discipline, and emotional support (Hammes et al., 2022; Pang et al., 2024; Shin et al., 2024). The central implication is therefore not that AI should be avoided, but that implementation should preserve coach expertise, athlete agency, referee accountability, and the coach–athlete relationship while using AI to make performance information more accessible and actionable.

## 6. Recommendations

**Figure 3:**
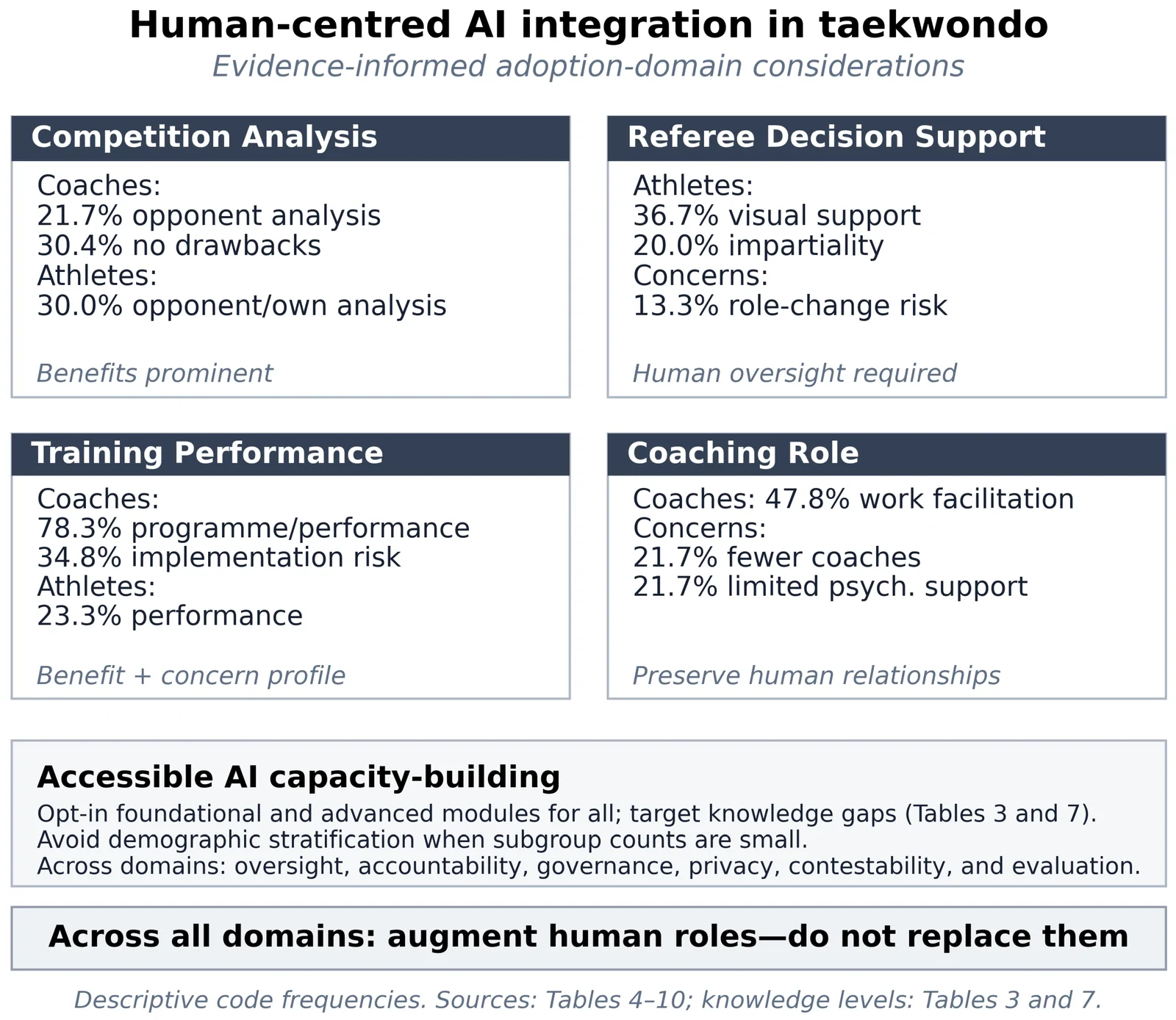
Evidence-informed considerations for human-centred AI integration in competitive taekwondo.

Based on the integrated findings, a set of evidence-informed considerations for human-centred AI integration in taekwondo is proposed, as illustrated in Figure 3.

Figure 3 summarises four adoption domains identified in the present sample and pairs them with cross-cutting capacity-building and human-role principles. Domain order is presentational only and does not imply implementation priority, sequencing, or deployment timeline. Percentages refer to descriptive code frequencies reported in Table 4, Table 5, Table 6, Table 7, Table 8, Table 9 and Table 10; they should not be read as readiness scores, prevalence estimates, or implementation priorities. The capacity-building component is intentionally framed for all participants rather than stratified by demographic category, given the exploratory nature of the within-sample knowledge patterns.

### Limitations

This study presents several limitations that should be considered when interpreting the findings. The research employed a non-probability convenience sampling approach with 53 participants from university taekwondo teams. As a result, the sample should not be treated as representative of taekwondo coaches, athletes, or martial arts practitioners more broadly, and the findings cannot support claims about prevalence or population-level attitudes. The focus on Turkish university taekwondo further limits transferability to recreational practitioners, professional or elite settings, other age groups, and cultural contexts in which AI adoption patterns, coaching relationships, and technology access may differ.

The qualitative nature of the research, while providing rich insights into participant perceptions, relies on self-reported data that may be subject to social desirability bias, recall limitations, or limited familiarity with AI capabilities among participants. The cross-sectional design captures perceptions at a single time point, potentially missing dynamic changes in attitudes as AI technologies evolve and become more prevalent in sports contexts. Self-reported AI knowledge levels were not validated against objective performance measures and should be treated as perceptions rather than as direct indicators of capability. Similarly, psychological readiness was not measured using a validated readiness, trust, self-determination, or self-efficacy instrument. It was inferred interpretively from coded qualitative accounts and therefore should be understood as an exploratory sensitising lens rather than as a validated psychological construct or measured outcome.

Interviews were conducted in Turkish and translated into English at the point of reporting. While coding was performed on the original Turkish transcripts and the translation workflow included bilingual review and machine-translation cross-checking, translation processes can attenuate idiomatic, emotional, and culturally specific nuances that are particularly material to interpretive qualitative analysis. The illustrative paraphrased statements presented in the Findings section should therefore be interpreted as condensed representations of coded meanings rather than as verbatim translated quotations. The use of a large language model as an auxiliary translation aid for the manuscript draft introduces a further dependency on a tool whose translation behaviour is not fully transparent; this is acknowledged as a methodological constraint despite the human review applied at each stage. Audio recording was relied upon for verbatim Turkish transcription, but in any case in which recording was not possible, contemporaneous note-taking may have introduced selective capture of participant statements.

The interview protocols for coaches and athletes were intentionally asymmetric in their final question (coaching-role evolution for coaches, referee decisions for athletes). This asymmetry was chosen to align each protocol with the domain most directly relevant to the participant group, but it restricts direct cross-stakeholder comparison on the coaching-role and referee domains. Differences between coach and athlete accounts in these domains may partly reflect protocol design rather than underlying stakeholder differences, and the dataset therefore cannot establish whether both groups would have responded similarly or differently if asked identical questions.

Gender analysis was exploratory, descriptive, and limited to binary categorisation, potentially overlooking more nuanced individual differences in technology acceptance patterns. The full sample and gender-disaggregated cell sizes are too small to support inferential, psychological, or population-level claims about gender differences. For the thematic code tables, gender is retained only as raw frequency counts and is not used to support subgroup claims. The within-gender percentages in the knowledge-level tables are likewise exploratory observations from this specific sample, not evidence of stable population-level differences. The study also examined perceptions of largely hypothetical or anticipated AI applications rather than actual deployed systems used by participants in training, competition analysis, coaching, or refereeing. Consequently, the findings cannot determine actual adoption behaviour, sustained trust, performance effects, usability, or unintended consequences that may emerge during real implementation. The manual coding process, despite inter-coder agreement procedures, remains subject to researcher interpretation, and the use of Cohen’s Kappa for multi-code data carries the low-prevalence and skewed-marginal limitations described in Section 3.4.

The thematic map presented in Figure 1 is heuristic and qualitative; placements are derived from relative prevalence patterns within the present sample rather than from validated measurement instruments, and they should not be used for inferential comparison, deployment timing, or priority ranking. Similarly, the set of considerations summarised in Figure 3 is descriptive: it consolidates participant-reported benefits and concerns across four adoption domains and pairs these with cross-cutting capacity-building and human-role principles. The figure does not propose validated readiness scores, implementation phases, or domain-priority rankings, and the capacity-building principle is intentionally framed for all participants rather than stratified by demographic category. Further empirical validation is required through prospective designs, field trials with actual AI tools, and purpose-built readiness instruments capable of testing whether the interpretive readiness patterns identified here are stable across settings. Finally, the study did not extensively examine the influence of factors such as coaching experience, athletic achievement level, or prior technology exposure on AI acceptance patterns, which could provide additional explanatory insights for implementation strategies.

## 7. Conclusions

In this qualitative case study, the findings suggest that university taekwondo coaches and athletes evaluate AI technologies through both opportunity-oriented and cautionary perspectives across training, competition analysis, coaching, and referee decision support. Coaches’ accounts suggest that AI may contribute to training performance, analysis processes, and data-informed decision-making, while also raising concerns about discipline, human relationships, and changes in professional roles. Athletes’ accounts suggest positive expectations regarding performance enhancement, personalised programme support, and more objective evaluation, alongside concerns about incorrect guidance and reduced emotional connection.

Overall, the findings suggest that AI may support taekwondo practice when it is positioned as an augmentative tool rather than as a replacement for human judgement. The human factor remains central because empathy, experience transfer, contextual judgement, athlete agency, and coach–athlete relationships were repeatedly identified as important to acceptable implementation. Ethical considerations, information reliability, transparency, and human oversight therefore warrant continued attention in future AI-supported taekwondo systems. The psychological readiness interpretation offered in this study should be treated as exploratory: self-determination theory and self-efficacy were used as sensitising lenses to interpret qualitative perceptions but not as validated measures or tested explanatory models. Further research should validate these readiness-related patterns with broader samples, purpose-built instruments, and studies of actual AI deployments in diverse taekwondo and martial arts settings.

## Figures and Tables

**Table 1:** Frequency (*f*) and percentage (%) distributions of coach participants’ demographic characteristics.

Variable	Category	*f*	%
Gender	Female	10	43.5
	Male	13	56.5
Coaching Experience	0–5 years	7	30.4
	6–10 years	5	21.7
	11–20 years	6	26.1
	21 years and above	5	21.7
University Count	Different Universities	19	82.6
	Same University	4	17.4

**Table 2:** Frequency (*f*) and percentage (%) distributions of athlete participants’ Demographic Characteristics.

Variable	Category	*f*	%
Gender	Female	14	46.7
	Male	16	53.3
Athletic Experience	0–5 years	5	16.7
	6–10 years	17	56.7
	11 years and above	8	26.7
Class Year	1st year	17	56.7
	2nd year	6	20.0
	3rd year	4	13.3
	4th year	3	10.0
University Count	Different Universities	22	73.3
	Same University	8	26.7

**Table 3:** Coaches’ self-reported artificial intelligence knowledge level by gender (descriptive within-sample percentages).

Knowledge Level	Codes (Illustrative Paraphrased Statements)	All Participants		Female		Male	
		*f*	%	*f*	%	*f*	%
No knowledge	I have no knowledge, I’ve only heard of it	5	21.7	3	30.0	2	15.4
Minimal knowledge	I have minimal knowledge, I’ve just learned about it	5	21.7	4	40.0	1	7.7
Moderate knowledge	I have moderate knowledge, I use ChatGPT	8	34.8	1	10.0	7	53.8
Good knowledge	I have good knowledge, I use Gemini	2	8.7	0	0.0	2	15.4
Usage mentioned (unclear level)	I use it frequently, I have knowledge	2	8.7	1	10.0	1	7.7
No response/Unclassifiable	—	1	4.3	1	10.0	0	0.0

Note: Female and male percentages are calculated within gender (female n=10, male n=13). All-participants percentages are calculated within all coaches (n=23). These values are exploratory within-sample descriptors only and are not suitable for inferential, psychological, or population-level claims about gender differences.

**Table 4:** Theme, sub-theme, code, overall frequency, percentage, and descriptive raw gender counts of coaches’ views on artificial intelligence effects on training performance.

Criteria	Sub-Theme	Codes (Illustrative Paraphrased Statements)	All *f*	All %	Female *f*	Male *f*
Benefits	Training Programme and Performance	Prepares training programmes, improves performance	18	78.3	8	10
	Data-based Support	Data-based training, athlete information, work facilitation	9	39.1	4	5
	Health and Motor Development	Health monitoring, development of fundamental motor characteristics	2	8.7	2	0
Drawbacks	Implementation Risks	Incorrect training programme, loss of discipline, laziness risk	8	34.8	3	5
	Coaching Role and Endurance	Coach relegated to background, negative impact on endurance	3	13.0	2	1

Note: Where a row reports one code, All % is the proportion of coach participants (n=23) mentioning the code at least once. Where a row groups multiple semantically related codes, *f* reports component-code mentions and the corresponding % reports those mentions divided by the relevant group *n*; these are not unique-participant counts or participant percentages. Female *f* and male *f* use the same row definition and are provided for transparency only, not for inferential, psychological, or population-level gender-difference claims. Multiple codes could be assigned per participant; totals may therefore exceed 100%.

**Table 5:** Theme, sub-theme, code, overall frequency, percentage, and descriptive raw gender counts of coaches’ views on artificial intelligence effects on competition analysis.

Criteria	Sub-Theme	Codes (Illustrative Paraphrased Statements)	All *f*	All %	Female *f*	Male *f*
Benefits	Technical and Operational Support	Systematic analysis, data recording/archive, score detection, coach support	7	30.4	1	6
	Opponent Analysis	Provides opponent analysis	5	21.7	3	2
	Error Detection	Sees unseen errors, detects incorrect techniques	3	13.0	2	1
	Performance Data Provision	Provides performance data (speed, strength, physiological measurement)	3	13.0	3	0
Drawbacks	Implementation and Competitive Risks	Incorrect information, opponents also using AI, time required for use	6	26.1	3	3
Neutral/Unclear	No Drawback or No Opinion	Sees no drawbacks, no opinion	8	34.8	2	6

Note: Where a row reports one code, All % is the proportion of coach participants (n=23) mentioning the code at least once. Where a row groups multiple semantically related codes, *f* reports component-code mentions and the corresponding % reports those mentions divided by the relevant group *n*; these are not unique-participant counts or participant percentages. Female *f* and male *f* use the same row definition and are provided for transparency only, not for inferential, psychological, or population-level gender-difference claims. Multiple codes could be assigned per participant; totals may therefore exceed 100%.

**Table 6:** Theme, sub-theme, code, overall frequency, percentage, and descriptive raw gender counts of coaches’ views on artificial intelligence effects on the future of the coaching role.

Criteria	Sub-Theme	Codes (Illustrative Paraphrased Statements)	All *f*	All %	Female *f*	Male *f*
Benefits	Work Facilitation	Facilitates coach’s work	11	47.8	5	6
	Professional Development and Information Support	Coach development, information provision, support for beginners	5	21.7	2	3
	Programme and Analysis Support	Training and nutrition programme support, more systematic analysis, perceived error reduction	4	17.4	0	4
Drawbacks	Relational and Psychological Concerns	Psychological/support deficiency, athlete-coach relationship deteriorates	7	30.4	4	3
	Role Security Concerns	Number of coaches may decrease	5	21.7	1	4
	Implementation Risks	Incorrect information risk, laziness risk	4	17.4	2	2
Neutral	No Opinion	No opinion	1	4.3	0	1

Note: Where a row reports one code, All % is the proportion of coach participants (n=23) mentioning the code at least once. Where a row groups multiple semantically related codes, *f* reports component-code mentions and the corresponding % reports those mentions divided by the relevant group *n*; these are not unique-participant counts or participant percentages. Female *f* and male *f* use the same row definition and are provided for transparency only, not for inferential, psychological, or population-level gender-difference claims. Multiple codes could be assigned per participant; totals may therefore exceed 100%.

**Table 7:** Athletes’ self-reported artificial intelligence knowledge level by gender (descriptive within-sample percentages).

Knowledge Level	Codes (Illustrative Paraphrased Statements)	All		Female		Male	
		*f*	%	*f*	%	*f*	%
No knowledge	I have no knowledge, I’ve only heard of it	9	30.0	4	28.6	5	31.3
Minimal knowledge	I have minimal knowledge, I’ve just learned about it	8	26.7	4	28.6	4	25.0
Moderate knowledge	I have moderate knowledge, I use ChatGPT	7	23.3	3	21.4	4	25.0
Good knowledge/regular use	I use it frequently, I have knowledge	6	20.0	3	21.4	3	18.8

Note: Female and male percentages are calculated within gender (female *n* = 14, male *n* = 16). All-participants percentages are calculated within all athletes (*n* = 30). These values are exploratory within-sample descriptors only and are not suitable for inferential, psychological, or population-level claims about gender differences.

**Table 8:** Theme, sub-theme, code, overall frequency, percentage, and descriptive raw gender counts of athletes’ views on artificial intelligence effects on training performance.

Criteria	Sub-Theme	Codes (Illustrative Paraphrased Statements)	All *f*	All %	Female *f*	Male *f*
Benefits	Performance Enhancement	Improves performance	7	23.3	3	4
	Training Planning Support	Training planning support	5	16.7	3	2
	Personalised Programme	Personalised/training-appropriate programme	5	16.7	5	0
	Nutrition Support	Nutrition/diet programme support	4	13.3	2	2
	Technical and Professional Support	Scientific data, analysis, and technical/electronic support	5	16.7	5	0
Drawbacks	Personal Adaptation and Human Fit	Personal adaptation deficiency, emotional/experience deficiency	5	16.7	2	3
	Incorrect Guidance Risk	Incorrect guidance/incorrect programme	3	10.0	2	1
	Safety and Productivity Risks	Overtraining risk, productivity reduction risk, inability to foresee injury risk	3	10.0	2	1
	Lack of Trust	Useless/lack of trust	2	6.7	0	2

Note: Where a row reports one code, All % is the proportion of athlete participants (n=30) mentioning the code at least once. Where a row groups multiple semantically related codes, *f* reports component-code mentions and the corresponding % reports those mentions divided by the relevant group *n*; these are not unique-participant counts or participant percentages. Female *f* and male *f* use the same row definition and are provided for transparency only, not for inferential, psychological, or population-level gender-difference claims. Multiple codes could be assigned per participant; totals may therefore exceed 100%.

**Table 9:** Theme, sub-theme, code, overall frequency, percentage, and descriptive raw gender counts of athletes’ views on artificial intelligence effects on competition analysis.

Criteria	Sub-Theme	Codes (Illustrative Paraphrased Statements)	All *f*	All %	Female *f*	Male *f*
Benefits	Opponent and Personal Analysis	Learning opponent’s/own strengths and weaknesses	9	30.0	5	4
	Deficiency Detection	Seeing deficiencies	4	13.3	3	1
	Strategy Development	Instant analysis/monitoring, strategy development	4	13.3	3	1
	Objective and Systematic Evaluation	Objective evaluation, penalty/scoring support, perceived error reduction	5	16.7	2	3
	Technical and Operational Support	Information support, physiological data, time saving	3	10.0	1	2
Drawbacks	Reliability and Information Risks	Incorrect guidance/incorrect analysis, information vulnerability	5	16.7	2	3
	Practical Relevance Concerns	Useless/ineffective	3	10.0	2	1

Note: Where a row reports one code, All % is the proportion of athlete participants (*n* = 30) mentioning the code at least once. Where a row groups multiple semantically related codes, *f* reports component-code mentions and the corresponding % reports those mentions divided by the relevant group *n*; these are not unique-participant counts or participant percentages. Female *f* and male *f* use the same row definition and are provided for transparency only, not for inferential, psychological, or population-level gender-difference claims. Multiple codes could be assigned per participant; totals may therefore exceed 100%.

**Table 10:** Criteria, theme, code, overall frequency, percentage, and descriptive raw gender counts of athletes’ views on artificial intelligence effects on referee decisions.

Criteria	Theme	Codes (Illustrative Paraphrased Statements)	All *f*	All %	Female *f*	Male *f*
Benefits	Visual Support	Seeing what referees cannot see	11	36.7	7	4
	Fairness, Decision Support, and Learning	Preventing bias, perceived referee-error reduction, transparent management, more just competition, referee education support	11	36.7	7	4
Drawbacks	Human Authority and Role Risks	Risk of eliminating refereeing role, authority/initiative reduction, AI dominance	8	26.7	3	5
	Match Management and Relational Risks	Management with AI problematic, referee-competition relationship	3	10.0	2	1

Note: Where a row reports one code, All % is the proportion of athlete participants (n=30) mentioning the code at least once. Where a row groups multiple semantically related codes, *f* reports component-code mentions and the corresponding % reports those mentions divided by the relevant group *n*; these are not unique-participant counts or participant percentages. Female *f* and male *f* use the same row definition and are provided for transparency only, not for inferential, psychological, or population-level gender-difference claims. Multiple codes could be assigned per participant; totals may therefore exceed 100%.

**Table 11:** Conceptual and analytical mapping of interview domains to readiness-related indicators and sensitising interpretations (not a validated measurement framework).

Interview/Coding Domain	Readiness-Related Indicator	Sensitising Interpretation
AI knowledge level	Familiarity with AI, perceived capability, and confidence-building needs	Self-efficacy and perceived competence; low self-reported knowledge is interpreted as a possible readiness barrier, not as an objective measure of ability
Training performance	Perceived usefulness of AI for programmes and performance, balanced against incorrect guidance, discipline loss, laziness, and adaptation concerns	Competence, autonomy, and trust; readiness is higher where AI is seen as augmenting training decisions and lower where it is seen as undermining control or capability
Competition analysis	Opponent/personal analysis, deficiency detection, strategy support, incorrect analysis, and trust concerns	Trust in AI outputs and perceived practical value; readiness depends on whether analysis support is seen as reliable and interpretable
Future coaching role	Work facilitation, role displacement, coach backgrounding, psychological support, and athlete–coach relationship concerns	Autonomy, role security, professional identity, and relatedness; readiness depends on preserving meaningful coaching authority and relational expertise
Referee decisions	Visual support, impartiality, human error reduction, authority reduction, role elimination, and AI dominance concerns	Procedural fairness, accountability, and human oversight; readiness depends on whether AI is framed as decision support rather than replacement

**Table 12:** Inter-coder agreement based on Cohen’s Kappa values, interpreted using Landis and Koch’s classification.

Coding Domain (Source Table)	Cohen’s Kappa	Landis and Koch (1977) Interpretation
Coach knowledge levels (Table 3)	0.865	Almost perfect agreement (0.81–1.00)
Coach training performance (Table 4)	0.718	Substantial agreement (0.61–0.80)
Coach competition analysis (Table 5)	0.882	Almost perfect agreement (0.81–1.00)
Future of the coaching role (Table 6)	0.778	Substantial agreement (0.61–0.80)
Athlete knowledge levels (Table 7)	0.697	Substantial agreement (0.61–0.80)
Athlete training performance (Table 8)	0.888	Almost perfect agreement (0.81–1.00)
Athlete competition analysis (Table 9)	0.872	Almost perfect agreement (0.81–1.00)
Athlete referee decisions (Table 10)	0.659	Substantial agreement (0.61–0.80)

## Data Availability

The data presented in this study are available on request from the corresponding author. The data are not publicly available due to privacy and ethical restrictions regarding the anonymity of the participants.
